# Dental Anomalies’ Characteristics

**DOI:** 10.3390/diagnostics11071161

**Published:** 2021-06-25

**Authors:** Tatiana Sella Tunis, Ofer Sarne, Israel Hershkovitz, Tamar Finkelstein, Aikaterini Maria Pavlidi, Yehoshua Shapira, Moshe Davidovitch, Nir Shpack

**Affiliations:** 1 Department of Anatomy and Anthropology, Sackler Faculty of Medicine, Tel Aviv University, Tel Aviv 69978, Israel; anatom2@tauex.tau.ac.il; 2 Dan David Center for Human Evolution and Biohistory Research, Shmunis Family Anthropology Institute, Sackler Faculty of Medicine, Tel Aviv University, Tel Aviv 69978, Israel; 3 Department of Orthodontics, The Maurice and Gabriela Goldschleger School of Dental Medicine, Tel Aviv University, Tel Aviv 69978, Israel; ofer@sarne.co.il (O.S.); tamar@vas.co.il (T.F.); ampavlidi@gmail.com (A.M.P.); yehoshua.shapira@gmail.com (Y.S.); moshedavidovitch@gmail.com (M.D.); nir@shpack.co.il (N.S.)

**Keywords:** dental anomalies, maxilla, mandible, growth and development, dental diagnosis

## Abstract

The aim of this study was to characterize dental anomalies. The pretreatment records (photographs and radiographs) of 2897 patients (41.4% males and 58.6% females) were utilized to detect dental anomalies. The dental anomalies studied were related to number, size and shape, position, and eruption. A Chi-square test was carried out to detect associations between dental anomalies, jaw, and sex. A total of 1041 (36%) of the subjects manifested at least one dental anomaly. The prevalence of all dental anomalies was jaw-dependent and greater in the maxilla, except for submerged and transmigrated teeth. The most frequently missing teeth were the maxillary lateral incisor (62.3%) and the mandibular second premolars (60.6%). The most frequent supernumerary teeth were the incisors in the maxilla (97%) and the first premolars in the mandible (43%). Dental anomalies are more frequent in the maxilla and mainly involve the anterior teeth; in the mandible, however, it is the posterior teeth. These differences can be attributed to the evolutionary history of the jaws and their diverse development patterns.

## 1. Introduction

Dental anomalies introduce significant esthetic and functional problems in both jaws [1]. Therefore, a thorough survey of the factors involved in their development is of major importance. Specific genetic factors have been reported to be responsible for the development of dental anomalies in each jaw [2,3]. In addition, disturbances created during tooth development may produce variations in the number of teeth (agenesis/supernumerary teeth) [4], their size and shape, and tooth bud position, which can affect both permanent and deciduous dentition of both jaws [5]. Since dental conditions are strongly associated with efficient food processing in the mouth, we would expect a strong selection against dental anomalies during human evolution—namely rare occurrences of missing or impacted teeth—because they could interfere with proper food digestion. This is true for most mammals, the great apes included, but not for humans, where the distribution of dental anomalies is reportedly common [6]. Since the development of teeth is a complex process carried out under strict molecular control [7], the ultimate question is why?

The maxilla and mandible house the upper and lower dentition, comprising the hard tissue functional unit of the masticatory apparatus. Despite a common function and a similar number of teeth in each jaw, the ontogeny and phylogeny of the maxilla and mandible differ, which may contribute to varied occurrences of dental anomalies in each jaw. The growth and development of the maxilla is distinct from the mandible because of its complex developmental interactions with other anatomical structures in the skull. Although the mandible is an isolated bone, formed by the junction of the right and left mandibular swellings at the midline, the maxillary right and left swellings develop and merge with the expanding medial limbs of the developing nasal swellings (part of the nasomaxillary complex), which is needed to form a continuous U-shaped arch [8].

Both jaws underwent considerable size and shape reduction during hominid evolution, which was accompanied by a similar reduction in tooth size [9]. Using morphometric analysis, Lieberman et al. [10] showed that anatomically modern *Homo sapiens* are uniquely characterized by their facial retraction. The encephalization process was associated with a general reduction in the splanchnocranium complex, which involved changes in the face, mandible, and the teeth [11]. A reduction in nasal protrusion was followed by a similar reduction in the upper jaw. Scott [12] suggested that the development of a cutaneous heat-regulating mechanism could have led to a significant reduction in and disuse of the nasal mechanism, resulting in nasomaxillary complex reduction. A dramatic reduction in the size of the mandible (mainly of the retromolar area) [13] during human evolution may be related to the development of dental anomalies.

The purpose of this study was to determine whether the prevalence and type of developmental dental anomalies are jaw-related. Considering the differences in the biological history (phylogeny and ontogeny) of the upper and lower jaw, we hypothesized the following: (1) the type of dental anomalies greatly varies regarding its prevalence; (2) dental anomalies are sex- and age-independent; (3) dental anomalies occurring in the maxilla will be found more frequently than those in the mandible due to the maxillary complex developmental process and the tight interaction with other cranial structures; and (4) dental anomalies will vary by tooth type.

## 2. Materials and Methods

### 2.1. Study Sample

This study was carried out using the initial (pretreatment) records of 2897 consecutively treated orthodontic patients of Caucasian origin: 1198 males (41.4%) and 1699 females (58.6%), aged 8 to 40 years old (mean age, 16.11 years; SD, 6.788 years). All medical records were taken between 1993 and 2017 at the Department of Orthodontics, the Maurice and Gabriela Goldschleger School of Dental Medicine, Tel-Aviv University, Israel. These records were maintained for diagnostic purposes unrelated to the present study. The research was approved by the institutional ethical review board of Tel Aviv University. Inclusion criteria were as follows: age > 8 years, complete initial orthodontic records, i.e., proper photographs of the dental arch, intact study models, and high-quality diagnostic radiographs. Exclusion criteria included the following: congenital cranio-facial disorders or syndromes, previous orthodontic treatment, previous surgery in the head and neck region, previous esthetic dental procedures, root canal treatment, a history of traumatic dental avulsion or previous extraction of permanent teeth, technically aberrant radiographs, and incomplete records.

### 2.2. Evaluation Procedure

For each patient, maxillary and mandibular dental arches were evaluated based on their initial records, which included: (1) intraoral photographs, (2) study models, and (3) panoramic and periapical radiographs. The records were examined to detect abnormalities in the permanent and deciduous teeth of both dental arches, excluding the third molars. The number of affected teeth and their location within each jaw were recorded.

### 2.3. Dental Anomalies

**Figure 1:**
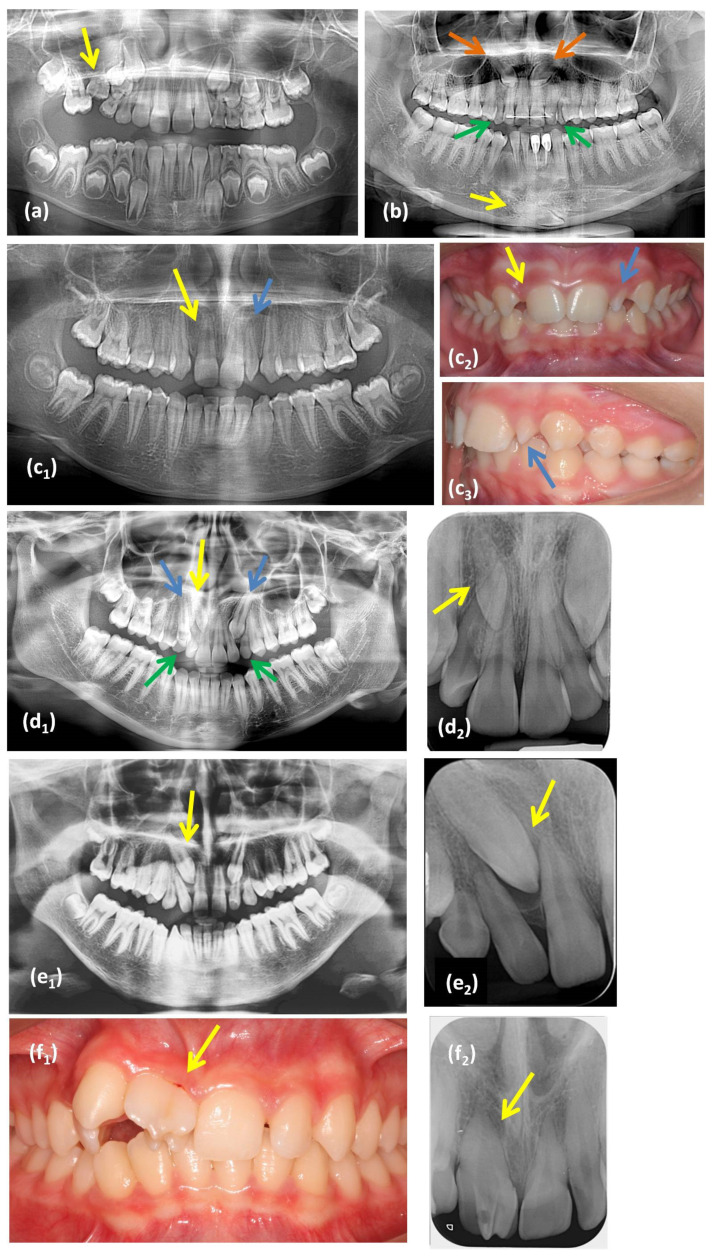
Examples of dental anomalies evaluated on radiographs and intraoral photographs: (**a**) panoramic radiograph of a patient with a submerged tooth #55 (yellow arrow); (**b**) panoramic radiograph of a patient with maxillary impacted canine teeth #23 and 13 (orange arrows), retained deciduous canine teeth #53 and 63 (green arrows), and a mandibular canine transmigration tooth #43 (yellow arrow) located at the lower border of the symphysis; (**c**) an example of a patient with a missing tooth #12 (yellow arrow) and a peg-shaped incisor tooth #22 (blue arrow): (**c_1_**) panoramic radiograph, (**c_2_**) intraoral frontal photograph, (**c_3_**) intraoral lateral photograph; (**d**) an example of a patient with a supernumerary tooth (impacted between the roots of the teeth #11–12, yellow arrow), impacted canine teeth #13 and 23 (blue arrows), and retained deciduous teeth #53 and 63 (green arrows): (**d_1_**) panoramic radiograph, (**d_2_**) periapical radiograph; (**e**) an example of a patient with dental transposition of the teeth #13 and #12 (yellow arrow): (**e_1_**) panoramic radiograph, (**e_2_**) periapical radiograph; (**f**) an example of a patient with fusion of the teeth #11 and 12 (yellow arrow): (**f_1_**) intraoral photograph, and (**f_2_**) periapical radiograph.

Eleven dental anomalies, divided into four categories (number, size and shape, position, and eruption), were considered in the study. Their diagnostic criteria appear in Table 1 and Figure 1.

### 2.4. Statistical Analysis

The data were recorded and analyzed using the SPSS software package (Statistical package for Social Sciences, version 20.0, SPSS, Inc., Chicago, IL, USA). Descriptive statistics, a frequency table, and pie charts were created for a general description of the dental anomalies in both jaws. A Chi-square test was carried out to detect a significant association between dental anomalies, sex, and age (8–12 years; ≥13 years). The level of statistical significance was set at *p* < 0.05.

### 2.5. Reliability

The intra- and inter-tester reliabilities for each dental anomaly were calculated, using the Kappa test, to determine the ability to accurately replicate the diagnosis of dental anomalies. The dental radiographs of 20 individuals were examined twice by the same researcher (T.S.T.) (two weeks apart and blinded to the initial screening results), and by an additional independent researcher (N.S.).

## 3. Results

### 3.1. Reliability Analysis

The Kappa values obtained showed high reproducibility for dental anomaly evaluation, ranging between 0.875 and 1.000.

### 3.2. Dental Anomalies: General Prevalence

Of the population studied (2897), more than one third (1041: 36%) manifested at least one of the eleven listed dental anomalies. The prevalence of each dental anomaly studied appears in Table 2. Tooth impaction (14.9%) was the most frequent anomaly, and fusion/gemination and transmigration were the rarest (0.3% each). For half of the studied anomalies, the prevalence was less than 1%. These included supernumerary teeth, fusion/gemination, transposition, transmigration, and submerged teeth. At least one missing tooth was evident in 9.3% of subjects; 11.9% of our subjects exhibited at least one retained primary tooth.

### 3.3. Dental Anomalies and Sex

The prevalence of the different dental anomalies (except for supernumerary teeth) was found to be similar in males (36.5%) and females (35.5%) (Table 2). Males were found to present the appearance of the supernumerary teeth more frequently, though the difference was small (1.3% in males vs. 0.4% in females).

### 3.4. Dental Anomalies and Age

No significant difference was found between the age groups regarding the prevalence of dental anomalies (*p* > 0.063), except for retained teeth, which were significantly more frequent in the older age group (*p* = 0.028) (Table 3).

### 3.5. Dental Anomalies and the Jaw

**Figure 2:**
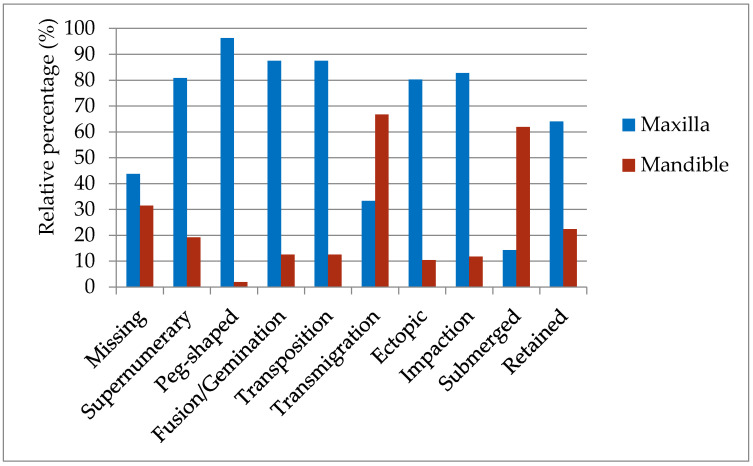
The relative frequencies of dental anomalies in the maxilla (blue) and mandible (red).

All dental anomalies, except for two, exhibited a greater prevalence in the upper jaw. Submerged teeth and transmigrated teeth exhibited a significantly higher prevalence in the lower jaw (62% vs. 14.3% and 66.7% vs. 33.3%, respectively) (Table 4, Figure 2). A considerable difference between the jaws was found with regard to peg-shaped teeth: 96.3% of the cases were recorded in the maxilla and only 1.9% in the mandible (1.8% presented in both jaws). Similarly, 82.8% of the impacted teeth were located in the maxilla, and only 11.8% were found in the mandible (5.4% in both jaws). Fusion/gemination and transposition prevalence were also much higher in the maxilla (87.5% of cases) compared with the mandible (12.5%), and most ectopically erupted teeth were found in the maxilla (80.2% of cases), with 10.4% in the mandible (9.4% in both jaws). Almost one quarter of the cases of missing and submerged teeth appeared in both jaws (Table 4). Five anomalies appeared only either on the upper or lower jaw (never in both): supernumerary, fusion/gemination, transposition, and transmigration cases.

### 3.6. Dental Anomalies and Tooth Type

**Figure 3:**
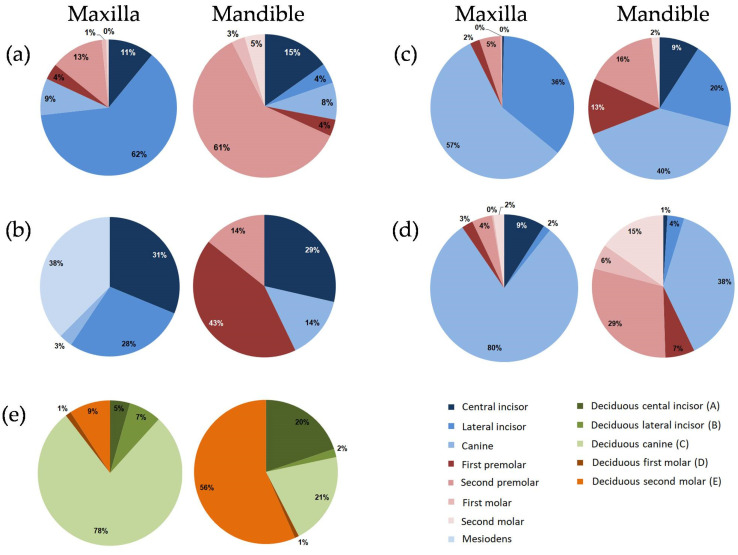
Distribution of dental anomalies by tooth type, jaw, and location (anterior (blue and green) vs. posterior (red and orange)): (**a**) missing teeth, (**b**) supernumerary teeth, (**c**) ectopic teeth, (**d**) impacted teeth, and (**e**) retained teeth.

Figure 3 shows the prevalence of dental anomalies by tooth type, jaw, and location.

Missing teeth: In the maxilla, the most frequently missing tooth was the lateral incisor (62.3% of all cases), and, in the mandible, it was the second premolar (60.61%) (Figure 3a).

Supernumerary teeth: In the maxilla, supernumerary teeth involved mainly the incisors (59.38% of all cases); mesiodens was the most frequent phenomenon (37.5%) (Figure 3b). In the mandible, 57.2% of the supernumerary cases involved the premolars, and only 28.6% involved the incisors. No cases of mesiodens were discovered in the mandible.

Peg-shaped teeth: In the maxilla, the majority of peg-shaped teeth involved the lateral incisor (97.5% of all cases); the remaining cases (2.5%) involved the canines. In the mandible, all peg-shaped teeth were the canines.

Fusion/gemination: In both jaws, these anomalies involved only the incisors.

Transposition: Most transpositions in the maxilla (85.7% of all cases) were associated with the canine: in half of the cases, it interchanged with the first premolar, and in 35.7% of the cases, it interchanged with the lateral incisor. In the mandible, all transpositions were between the canine and lateral incisor.

Transmigration: The canine was the only transmigrated tooth found in both jaws. In the maxilla, 2/3 of the transmigrated canines were found on the left side and 1/3 on the right side; in the mandible, the opposite was observed (2/3 on the right and 1/3 on the left side).

Ectopic teeth: The canine was found to be the most common ectopic tooth in both jaws, followed by the lateral incisor and the second premolar (Figure 3c).

Impacted teeth: In the maxilla, the canines comprised the majority of the impacted teeth (80% of all cases), followed by the central incisors (9%) (Figure 3d). In the mandible, the canine was the most commonly impacted tooth (38.1%), followed by the second premolar (29.5%). The central incisor was the least impacted tooth in the mandible (1% compared with 9% in the maxilla).

Submerged teeth: In both jaws, the second deciduous molars were the most affected teeth (100% and 95% in the maxilla and mandible, respectively).

Retained teeth: In the maxilla, the deciduous canine was the most commonly retained tooth (77.8% of all upper jaw cases), whereas, in the mandible, the retained canine was found only in 20.8% of the cases (Figure 3e). The second deciduous molar was the most commonly retained tooth in the mandible (56.6%), compared with only 9.2% in the maxilla.

### 3.7. Dental Anomalies and Location (Anterior vs. Posterior)

Data on the location (anterior teeth vs. posterior teeth) of dental anomalies by jaws appear in Table 5. In the maxilla, all anomalies, except submerged teeth, appeared in the anterior region, ranging from 82% to 100%. In the mandible, however, this was found to be more complex: half of the anomalies (missing, supernumerary, impaction, submerged, and retained teeth) appeared mostly in the posterior region (range: 57.1%–100%); the other anomalies (peg-shaped teeth, fusion/gemination, transmigration, and ectopic teeth) appeared mainly in the anterior region (range: 69.1–100%).

## 4. Discussion

### 4.1. Demography and Dental Anomalies

In the current study, we found that approximately one third of the subjects studied manifested at least one dental anomaly. Tooth impaction was the most common dental anomaly (14.9%), followed by ectopic tooth eruption (14.3%) and a retained tooth (11.9%). Missing teeth were found in 9.3% of the cases. No significant differences were found in the prevalence of dental anomalies between males and females, except for supernumerary teeth. In our study, males had a significantly greater prevalence of supernumerary teeth (1.3%) compared with females (0.4%), which is in agreement with previous reports [21]. Similarly, Bilge et al. [22] found at least one dental anomaly in 39.2% of the subjects that they studied, with no significant difference between the sexes, and they reported tooth impaction as the most prevalent anomaly (17.83%).

### 4.2. Jaws and Dental Anomalies

All dental anomalies discovered in the present study were found to be more frequent in the maxilla than the mandible, except for transmigrated and submerged teeth (Figure 2). Differences in the prevalence of certain dental anomalies between the upper and lower jaws have been previously reported. For example, Celikoglu et al. [23] showed that maxillary canines were impacted in 4.9% of orthodontic subjects, whereas mandibular impacted canines were observed in only 0.4%. Similar to our findings, they also reported that canine transmigration was more frequent in the lower jaw (5 out of 6 cases). In a succeeding study [24], they noted that tooth agenesis was more frequent in the maxilla (60.2%) than in the mandible (39.8%), as was missing permanent canines (58% vs. 42%). Although not included in the present study, Kazanci et al. [25] and Dachi et al. [26] found that significantly more third molar teeth were missing in the maxilla than in the mandible, with a ratio of approximately 1.5:1.

### 4.3. Location and Dental Anomalies

The literature does not contain reports relating to dental anomalies by location. In the present study, dental anomalies in the upper jaw were mainly found in the anterior teeth and in the lower jaw, the posterior teeth. This dichotomous distribution and overall frequency of occurrence requires explanation of their disproportionate appearance in humans.

Why are dental anomalies so common? It is indeed remarkable that every third individual will present with some form of dental anomaly, a rate not seen in any other taxa. The human masticatory system has undergone a rapid and considerable reduction in size and orientation as a result of evolutionary changes [10,11], which are still ongoing [27,28]. This continuous pressure on the masticatory system to adapt to changing demands is likely related to the frequency in the occurrence of dental anomalies in humans. Lavelle and Moore [6] compared the frequency of missing and supernumerary teeth between monkeys, great and lesser apes, and humans, and found a significantly greater frequency of missing teeth in humans (11.1% vs. 1% in great apes), whereas supernumerary teeth were more frequently found in great apes (6.2% vs. 1.9% in humans). The authors suggested that this finding is related to the development of a shortened maxillo-mandibular skeleton in *Homo sapiens* compared with their earlier ancestors.

Why the upper jaw? The higher prevalence of dental anomalies in the maxilla, compared with the mandible, is probably partially due to the more complex development of the upper jaw: the lower jaw’s development is independent of the rest of the facial bones, whereas the maxilla’s development is not. It is therefore not surprising that an association between dental anomalies (e.g., a missing lateral incisor) and orofacial clefts was found [29]. A lack of fusion between the maxillary and medial nasal prominences (in order to create a primary palate) could result in insufficient mesenchyme to support the formation of tooth buds, which impacts the development of tooth agenesis [29,30]. Supernumerary teeth can also be developed in proximity to orofacial cleft as a result of dental lamina hyperactivity or tooth bud division [29,31]. None of these latter phenomena are associated with mandibular structures, hence the relatively lower frequency of related anomalies.

Why are the anterior teeth in the maxilla and the posterior teeth in the mandible more affected? In the present study, the majority of dental anomalies found in the maxilla were discovered in the anterior region, but those in the mandible were found predominantly in the posterior areas. For example, nearly 2/3 of missing teeth were the lateral incisors in the maxilla, and the second premolars in the mandible (Figure 3). Moreover, all the cases of supernumerary teeth in the maxilla involved the incisors (97%) and canines (3%), whereas, in the mandible, more than half of the supernumerary teeth involved the first and second premolars (57.2%). Similarly, Finkelstein et al. [32] reported that 84% of the supernumerary teeth were in the anterior region of the maxilla, and only 16% in the mandible. Other studies also reported that the majority of the supernumerary teeth involved the incisor in the maxilla (the anterior region) and the premolars (the posterior region) in the mandible [21,33]. Al-Abdallah et al. [34] reported that tooth agenesis isolated in the maxilla was associated with microdontia of the maxillary lateral incisors, whereas in the mandible, it was associated with retained and infraoccluded deciduous molars or impacted teeth. Lavelle and Moore [6] found that agenesis of the third molars was nearly equally distributed in both the maxilla and mandible, whereas agenesis in the incisors was limited to the maxilla and agenesis in the premolars was limited to the mandible. These findings support those found in the present study.

Congenitally missing teeth were previously proposed to result from phylogenetic evolution, in which a reduction in both the number and the size of the teeth occurred together with a decrease in jaw size [9]. This is contrasted with the appearance of the supernumerary teeth, which has been explained as resulting from atavism (phylogenetic reversion), which is the reappearance of an ancestral condition [21].

In our study, tooth impaction was the most frequently found dental anomaly: it was nearly seven times more common in the maxilla than in the mandible. The majority of the impacted maxillary teeth consisted of the canines (80%) and the central incisors (9%). When detected in the mandibular dentition, the majority of the impactions were discovered in the posterior region (57.1%). Previous studies reported that the incidence of tooth impaction varies from 6.1% to 18.2% of the population [22,35,36]. Hou et al. found that the incidence of tooth impaction, based on a panoramic evaluation of dental Chinese patients, was 6.15% [36]. Bilge et al. found that tooth impaction occurred in 17.83% of Turkish dental patients, according to their panoramic radiographs [22]. Kramer and Williams found at least one dental impaction in 18.2% of African American patients who visited an oral surgery clinic [35]. The maxillary permanent canine was found to be the most common impaction (excluding the third molars) [37]. Maxillary canine impaction has been reported to be 20 times higher than mandibular canine impaction [38]. In the current study, we noted that, although the maxillary canines are significantly more frequently impacted, the probability of their transmigration is much higher in the mandible. All of the transmigrated teeth found in our study comprised maxillary and mandibular canines. Canine transmigration was a relatively rare dental anomaly (0.3% prevalence), yet a significant difference between the jaws was found. Mandibular canine transmigration was two times more frequent than maxillary canine transmigration (Table 4). Owing to the nature of mandibular complete fusion at the symphysis, the lower impacted canines are free to cross to the opposite side of the arch. This in contrast to the midpalatal suture that exists in the maxilla, which possibly presents a physical barrier for tooth migration across the midline (the right and left maxilla are two separate bones).

In addition, we found that the most frequently retained tooth in the maxilla is the deciduous canine (78%), whereas, in the mandible, it is the second deciduous molar (57%). Local factors such as prolonged retention of deciduous teeth and supernumerary teeth have been reported as possible factors contributing to the impaction of the permanent teeth [36,37]. Lappin [39] suggested that non-resorption of the deciduous canine root may cause permanent canine impaction. Clinical reports have shown that timely deciduous canine extraction, in the case of potential maxillary permanent canine impaction, indeed appears to encourage its spontaneous eruption [40]. In addition, there may be an inverse relationship, in that impaction of the permanent maxillary canine or mandibular second premolar may permit retention of their primary analogs.

Interestingly, all of these local direct factors explain specific anomalies; however, they do not explain why the posterior teeth in the mandible and the anterior teeth in the maxilla exhibit higher rates of anomalies. A possible explanation lies in the differences in jaw ontogenesis. The arch space for the deciduous molars is created mainly by an increase in the length of the posterior region and is determined by the age of 1 to 2 years. Liu et al. [41] found that ramus growth and remodeling are greatest during the age of 0.4–1 year, whereas mandibular symphysis exhibits no significant remodeling changes. Between 2 and 5 years of age, the mandibular growth rates significantly decrease, which is probably related to the completion of deciduous teeth eruption. In a longitudinal study by Bishara et al. [42], it was found that the greatest incremental increase in the mandibular arch length was during the first two years of life. The mandibular body grows longer mainly by bone apposition on the posterior surface of the ramus, and resorption occurs at the anterior surface [8]. In infancy, the mandibular ramus is located where the primary first molar will eventually erupt, and it relocates posteriorly through the process of remodeling, which creates space for the second primary molar and the permanent molar teeth [43]. Insufficient remodeling of the ramus on its anterior and posterior borders could interfere with the availability of space for eruption of the posterior permanent teeth and could increase the chances of dental anomalies in these areas. For example, Al-Gunaid et al. [44] found significantly smaller retromolar space in individuals with impacted lower third molars compared with controls. Several authors [4,45] previously reported that certain regions during tooth development are more susceptible to epigenetic influences, such as the maxillary lateral incisor, which develops in the area of the embryonic fusion between the lateral maxillary and medial nasal processes. In the mandible, permanent tooth agenesis occurs most frequently in the area of the second premolar, which is associated with the distal end of the primary dental lamina. A further site of tooth agenesis is in the area where the mandibular processes fuse at the midline and is where the lower central incisors develop. Moreover, a previous investigation showed that delayed dental development is associated with hypodontia [46]. The greatest delay was found in the development of the mandibular second premolars, followed by the mandibular first and second molars, whereas no significant differences were found for the incisors, canines, and first molars. Decreased arch length due to jaw size reduction, combined with delayed development of posterior teeth, may explain the increased susceptibility to the occurrence of dental anomalies in this area.

## 5. Limitations of the Study

Although the study population was heterogeneous, generalization of the results requires further study of populations of different geographical origin. Similarly, the association between dental anomalies and their distribution within the jaws needs further confirmation and validation from other populations. Deducing data on the development of dental anomalies from the current study sample were limited to orthodontic patients, which justifies further investigation of the general population. Future studies should examine the possible confounding effect of socioeconomic factors on dental anomalies. Additionally, the findings of the present study clearly show prevalence differences indental anomalies between the jaws and their different locations within each jaw. However, establishing the role of evolutionary processes requires further anthropological studies on dental anomalies in primates and early and late *Homo* populations.

## 6. Conclusions

The prevalence and severity of dental anomalies are high in human populations, and they are jaw- and location-dependent. Most dental anomalies occurring in the upper jaw involve the anterior region, whereas the inverse was found in the mandible. Dental anomalies are sex- and age-independent. The high rate of dental anomalies and the differences between the jaws can be explained by differences in their evolutionary history and ontogeny.

## Figures and Tables

**Table 1:** Dental anomalies considered in the current study and their diagnostic criteria.

Dental Anomaly		Diagnostic Criteria
Category	Name	
Number	Missing teeth	The lack of development of one or more permanent teeth.
	Supernumerary teeth	An increase in the normal number of permanent teeth due to the development of additional teeth.
Size and shape	Peg-shaped teeth	A tooth with incisal mesio-distal width smaller than its cervical width [14].
	Fusion	Union between two separate tooth buds during dental development involving the crowns and/or the roots [15].
	Gemination	Incomplete division of a tooth germ, resulting in the formation of two partially or completely separated crowns with one root and one root canal. It is clinically characterized by incisal notching on an enlarged crown [15].
Position	Transposition	Interchange in the position of two adjacent permanent teeth in the same quadrant of the dental arch [16].
	Transmigration	Movement of an impacted tooth across the jaw midline [17].
Eruption	Ectopic	An erupted tooth that is not in its proper position in the dental arch (e.g., the tooth is located mesially/distally [18] or vestibularly/orally outside the dental arch.
	Impaction	Cessation of the eruption of a tooth caused by a clinically or radiographically detectable physical barrier in the eruption path, or because of an abnormal position of the tooth [19,20].
	Submerged	A deciduous erupted tooth that failed to reach the occlusal level of the fully erupted adjacent teeth by at least 2 mm.
	Retained	Failure of the primary tooth to exfoliate at the proper developmental stage (more than one year late to erupt of its permanent successor).

**Table 2:** Prevalence of dental anomalies by sex.

Dental Anomaly	Sex	*n*	Prevalence (%)	*p*-Value ^1^
Missing	Male	118	9.8	0.41
	Female	152	8.9	
	Total	270	9.3	
Supernumerary	Male	19	1.3	**0.001**
	Female	7	0.4	
	Total	26	0.9	
Peg-shaped	Male	22	1.8	0.927
	Female	32	1.9	
	Total	54	1.9	
Fusion/Gemination	Male	4	0.3	0.619
	Female	4	0.2	
	Total	8	0.3	
Transposition	Male	3	0.3	0.066
	Female	13	0.8	
	Total	16	0.6	
Transmigration	Male	3	0.3	0.625
	Female	6	0.4	
	Total	9	0.3	
Ectopic	Male	177	14.8	0.53
	Female	237	13.9	
	Total	414	14.3	
Impaction	Male	174	14.5	0.654
	Female	257	15.1	
	Total	431	14.9	
Submerged	Male	8	0.7	0.761
	Female	13	0.8	
	Total	21	0.7	
Retained	Male	151	12.6	0.308
	Female	193	11.4	
	Total	344	11.9	

^1^*p*-values for the difference between the sexes. Significant values are denoted in bold.

**Table 3:** Prevalence of dental anomalies by age groups (up to 12 years old, *n* = 1074; and above the age of 13, *n* = 1823).

Dental Anomaly	Age Group	*n*	Prevalence (%)	*p*-Value ^1^
Missing	8–12 y	88	8.2	0.109
	≤13 y	182	10.0	
Supernumerary	8–12 y	13	1.2	0.170
	≤13 y	13	0.7	
Peg-shaped	8–12 y	15	1.4	0.153
	≤13 y	39	2.1	
Fusion/Gemination	8–12 y	4	0.4	0.448
	≤13 y	4	0.2	
Transposition	8–12 y	6	0.6	0.972
	≤13 y	10	0.5	
Transmigration	8–12 y	1	0.1	0.106
	≤13 y	8	0.4	
Ectopic	8–12 y	151	14.1	0.776
	≤13 y	263	14.4	
Impaction	8–12 y	177	16.5	0.063
	≤13 y	254	13.9	
Submerged	8–12 y	6	0.6	0.418
	≤13 y	15	0.8	
Retained	8–12 y	109	10.1	**0.028**
	≤13 y	235	12.9	

^1^*p*-values for the difference between the age groups. Significant values are denoted in bold. y = years.

**Table 4:** The relative frequencies of dental anomalies in the upper and lower jaws.

Dental Anomaly	Maxilla		Mandible		Both Jaws	
	*n*	%	*n*	%	*n*	%
Missing	118	43.7	85	31.5	67	24.8
Supernumerary	21	80.8	5	19.2	0	0
Peg-shaped	52	96.3	1	1.9	1	1.9
Fusion/Gemination	7	87.5	1	12.5	0	0
Transposition	14	87.5	2	12.5	0	0
Transmigration	3	33.3	6	66.7	0	0
Ectopic	332	80.2	43	10.4	39	9.4
Impaction	357	82.8	51	11.8	23	5.4
Submerged	3	14.3	13	61.9	5	23.8
Retained	220	64.0	77	22.4	47	13.7

**Table 5:** The relative frequencies of dental anomalies according to their anterior and posterior location.

Dental Anomaly	Maxilla		Mandible	
	Anterior	Posterior	Anterior	Posterior
	Relative Frequency (%)		Relative Frequency (%)	
Missing	82.0	18.0	28.0	72.0
Supernumerary	100.0	0.0	42.9	57.1
Peg-shaped	100.0	0.0	100.0	0.0
Fusion/Gemination	100.0	0.0	100.0	0.0
Transmigration	100.0	0.0	100.0	0.0
Ectopic	92.6	7.4	69.1	30.9
Impaction	90.4	9.6	42.9	57.1
Submerged	0.0	100.0	0.0	100.0
Retained	89.5	10.5	42.5	57.5

## Data Availability

The data sets analyzed during the current study are available from the corresponding author on request.
